# Genomic Prediction in a Multiploid Crop: Genotype by Environment Interaction and Allele Dosage Effects on Predictive Ability in Banana

**DOI:** 10.3835/plantgenome2017.10.0090

**Published:** 2018-07-01

**Authors:** Moses Nyine, Brigitte Uwimana, Nicolas Blavet, Eva Hřibová, Helena Vanrespaille, Michael Batte, Violet Akech, Allan Brown, Jim Lorenzen, Rony Swennen, Jaroslav Doležel

**Affiliations:** ^1^ Faculty of Science Palacký University CZ‐77146 Olomouc Czech Republic; ^2^ International Institute of Tropical Agriculture 7878 Kampala Uganda; ^3^ Institute of Experimental Botany, Centre of the Region Haná for Biotechnological and Agricultural Research CZ‐78371 Olomouc Czech Republic; ^4^ Lab. of Tropical Crop Improvement Division of Crop Biotechnics Katholieke Universiteit 2455 3001 Leuven Belgium; ^5^ International Institute of Tropical Agriculture 7878 Kampala Uganda; ^6^ Present address: Bill and Melinda Gates Foundation 23350 Seattle; ^7^ International Institute of Tropical Agriculture 10 Arusha Tanzania

## Abstract

Improving the efficiency of selection in conventional crossbreeding is a major priority in banana (*Musa* spp.) breeding. Routine application of classical marker assisted selection (MAS) is lagging in banana due to limitations in MAS tools. Genomic selection (GS) based on genomic prediction models can address some limitations of classical MAS, but the use of GS in banana has not been reported to date. The aim of this study was to evaluate the predictive ability of six genomic prediction models for 15 traits in a multi‐ploidy training population. The population consisted of 307 banana genotypes phenotyped under low and high input field management conditions for two crop cycles. The single nucleotide polymorphism (SNP) markers used to fit the models were obtained from genotyping by sequencing (GBS) data. Models that account for additive genetic effects provided better predictions with 12 out of 15 traits. The performance of BayesB model was superior to other models particularly on fruit filling and fruit bunch traits. Models that included averaged environment data were more robust in trait prediction even with a reduced number of markers. Accounting for allele dosage in SNP markers (AD‐SNP) reduced predictive ability relative to traditional bi‐allelic SNP (BA‐SNP), but the prediction trend remained the same across traits. The high predictive values (0.47– 0.75) of fruit filling and fruit bunch traits show the potential of genomic prediction to increase selection efficiency in banana breeding.

**Abbreviations**
AD‐SNP: allele dosage single nucleotide polymorphism
BA‐SNP: bi‐allelic single nucleotide polymorphism
BL: Bayesian least absolute shrinkage and selection operator
BLUP: best linear unbiased prediction
BRR: Bayesian ridge regression
cv.: cultivar
EAHB: East African highland banana
GATK: genome analysis toolkit
GBS: genotyping by sequencing
GEBV: genomic estimated breeding value
GS: genomic selection
GS1: genomic selection trial one
GS2: genomic selection trial two
IITA: International Institute of Tropical Agriculture
LIP: loss in predictive ability
MAS: marker assisted selection
NPK: nitrogen; phosphorus and potassium
PDP: percentage difference in prediction
PHF: plant height at flowering
PLP: percentage loss in prediction
QTL: quantitative trait loci
RKHS_M: reproducing kernel Hilbert space with markers
RKHS_P: reproducing kernel Hilbert space with pedigree
RKHS_PM: reproducing kernel Hilbert space with pedigree and markers
SNP: single nucleotide polymorphism

## Core Ideas

- First empirical evidence of genomic prediction in a multi‐ploidy banana population is presented.
- The effect of allele dosage single nucleotide polymorphism on prediction accuracy depends on the trait.
- Use of averaged environmental data improves prediction accuracy.
- BayesB model can be used across all traits during genomic prediction in banana breeding.
- The high predictive values show the potential of genomic prediction in banana breeding.

Bananas are large, perennial, herbaceous monocots with a majority of cultivated types being triploid (2*n* = 3*x* = 33). They are a staple food to millions of people in many tropical countries and a source of income for many homesteads. Triploid bananas are mostly sterile although some cultivars have residual fertility that leads to limited seed production when hand pollinated (Ssebuliba et al., 2006). They are vegetatively propagated by means of suckers, a method that limits gene flow and recombination. The lack of genetic variability of bananas grown in particular regions renders all cultivars susceptible to pests, pathogens and environmental stress. This causes reduced productivity of bananas that leads to food insecurity and income loss.

**Fig. 1:**
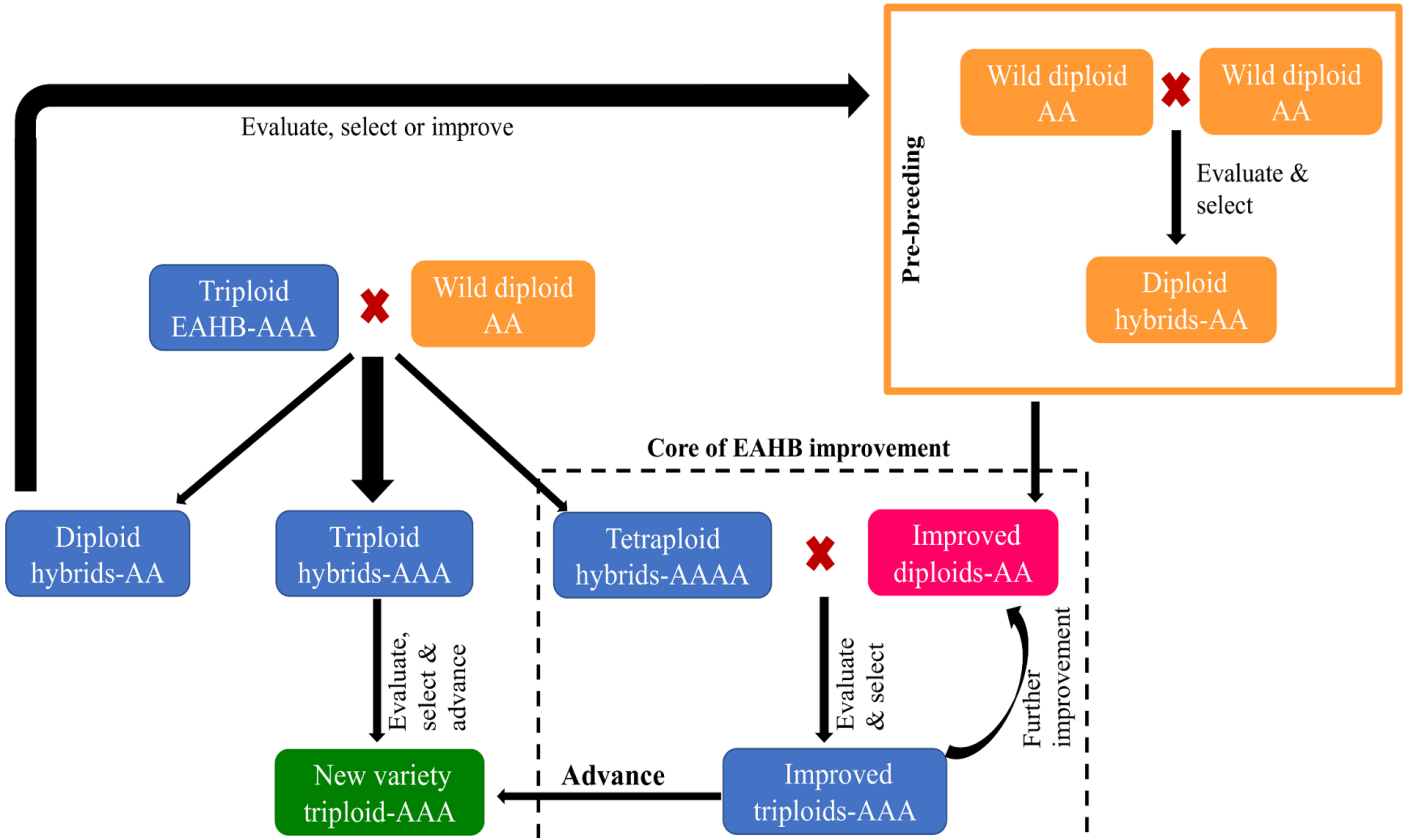
Conventional crossbreeding of East African Highland bananas (EAHB) starts with crossing a triploid parthenocarpic landrace with a wild, seeded diploid accession or a diploid cultivar showing fruit parthenocarpy. This cross gives diploids, triploids and tetraploid hybrids. Tetraploids are selected and crossed with improved diploid hybrids selected from inter‐diploid crosses. The resulting secondary triploids are evaluated, selected and advanced as promising improved genotypes aiming at new cultivars. The diploid and triploid (if fertile) hybrids can be further improved by crossing with other wild or improved diploids.

**Fig. 2:**
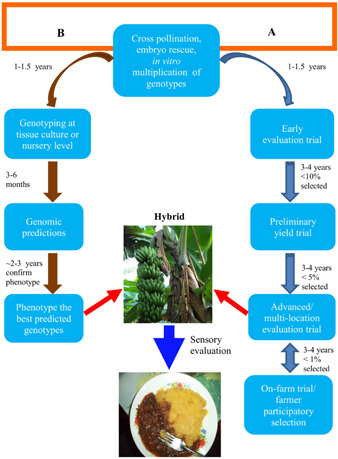
Approaches to hybrid selection in banana breeding program. (A) The classical phenotypic selection of banana hybrids and (B) integrated genomic selection and phenotypic selection approach being investigated.

Given the importance of banana, improving the resistance of cultivated bananas is the most sustainable solution to declining production (Simmonds, 1986; Rowe, 1990). This can be achieved by crossing with wild or improved diploids that carry host plant resistance genes for pathogens and pests. The triploid nature of cultivated bananas such as the East African highland banana (EAHB), impedes the breeding process due to low fertility or complete sterility of most cultivars. To overcome this problem, breeders have to develop intermediary improved diploids and tetraploids, which serve as parents to generate secondary triploids that are resistant and high yielding. Unlike a majority of crops, banana breeding involves crossing parents of different ploidy levels (Fig. 1). Partial fertility of polyploids relies on irregular meiosis and progenies consist of individuals with different ploidy. Due to linkage drag of undesirable alleles, several evaluations and phenotypic selection at various stages are implemented making banana breeding (depicted in Fig. 2) expensive and slow. Clearly, the integration of molecular tools into conventional breeding programs is required to increase banana breeding efficiency.

Marker assisted selection (MAS) helps in selection of genotypes carrying the trait of interest at an early stage. However, very few reports on the use of MAS in banana improvement are available. For example, markers have been used to screen for *Fusarium* tropical race 4 resistance and identification of banana hybrids that are devoid of infectious endogenous banana streak virus in the B‐genome (Wang et al., 2012b; Umber et al., 2016; Noumbissié et al., 2016). Most MAS technologies aim at identifying molecular markers that are linked to traits through quantitative trait loci (QTL) analysis. Once the markers are identified, the breeder can use them to track the inheritance of the traits of interest. Marker assisted selection has been successfully implemented where traits are controlled by a few QTL with major genetic effects (Asíns, 2002; Collard and Mackill, 2008). However, some traits such as yield, drought tolerance, and some others may be controlled by numerous QTL, each explaining a small portion of the genetic variance (Asíns, 2002). Identifying all QTL controlling such traits and the markers that are in linkage disequilibrium with those QTL becomes a challenge. Even if it would be possible to identify small‐effect QTL, their introgression into active breeding programs would be extremely challenging.

A relatively new approach of MAS in plant breeding known as genomic selection (GS) that uses genomic prediction models was proposed by Meuwissen et al. (2001). Several variants of the original GS methodology have also been proposed (Goiffon et al., 2017). In GS, high‐density markers spread across the entire genome are utilized to estimate the genetic value of a genotype using statistical models. As this estimate is based on genomic data, it is referred to as genomic estimated breeding value (GEBV). The primary advantage of GS over other forms of MAS is that the identification of individual QTL associated with a trait of interest is not necessary because QTL are assumed to be in linkage disequilibrium with at least one or more SNP (Meuwissen et al., 2001; Desta and Ortiz, 2014). The decrease in genotyping costs by next generation sequencing technologies and the emergence of GBS, which allows SNP discovery in large populations, made genomic prediction possible (Elshire et al., 2011). As the generation of marker data becomes increasingly cheaper than phenotyping, it is expected that GS will reduce breeding costs, increase selection intensity and accelerate the breeding efficiency.

Genomic selection is implemented in three phases that include: training, validation, and breeding (Jannink et al., 2010; Nakaya and Isobe, 2012). In the training phase, a model of the form “predicted phenotype = general phenotype mean in the population (intercept) + GEBV + residual error” is generated from both phenotypic and genotypic data. The predictive ability of a genomic prediction model is determined by cross validation as the correlation between the predicted and observed value of a trait or the correlation between GEBV and observed phenotype (Jannink et al., 2010; Crossa et al., 2014; Crossa et al., 2016).

Genomic selection has been successful in animal breeding (Gorddard and Hayes, 2007). It is also expected to increase genetic gain per unit time and cost in plant breeding especially when applied on traits with low heritability for which phenotypic selection is difficult and for crops with long selection cycle such as fruit trees, or banana (Wong and Bernardo, 2008; Crossa et al., 2010; Beaulieu et al., 2014; Crossa et al., 2014). Different studies in plants and animals have tested the predictive ability, or accuracy of different genomic prediction models (Legarra et al., 2008; Heffner et al., 2011; Kumar et al., 2012; Würschum et al., 2013; Crossa et al., 2016; Weng et al., 2016; Momen et al., 2017). These include best linear unbiased prediction (BLUP) and different Bayesian models (Robinson, 1991; Tibshirani, 1996; Meuwissen et al., 2001; Park and Casella, 2008; Zhang et al., 2010; Pérez and de los Campos, 2014). Characteristics of the models are summarized in numerous publications (Meuwissen et al., 2001; Habier et al., 2011; Desta and Ortiz, 2014; Pérez and de los Campos, 2014). Although these models were originally developed and optimized for diploid organisms, they have then been extended to polyploid organisms (Crossa et al., 2014; Gezan et al., 2017). However, all studies used populations with organisms of the same ploidy level. Polyploid organisms are challenging to model due to (i) uncertainty of allele frequency in the population and (ii) uncertainty of allele dosage at the loci (Blischak et al., 2016).

For bananas, besides the polyploid nature, there is a small effective breeding population. Yet the accuracy of genomic prediction depends on the size of training population. It should be large enough to capture all the segregating alleles in the breeding genetic pool (Crossa et al., 2014; Bassi et al., 2016). However, as noted by Bassi et al. (2016), no ideal population size exists for all species and traits. The parameters that need to be considered include relatedness of the individuals, the heritability of the trait, differences in linkage disequilibrium between markers and QTL across training and breeding populations, whether the population is bi‐parental, or a mixture of several families and the cost involved in phenotyping the training population. For example, Beaulieu et al. (2014) used 1694 open pollinated genotypes of white spruce with 6385 SNP markers and obtained different accuracies of prediction depending on the trait and the relationship between the training and validation data sets. The highest predictive ability observed was 0.44 for cell radial diameter. In contrast, Crossa et al. (2010) used a maize population of less than 300 individuals with less than 1200 markers and obtained a predictive ability as high as 0.79 for male flowering under well‐watered conditions.

This study explored the potential of genomic prediction in banana, a polyploid crop for which the population was composed of individuals with different ploidy levels, but mostly triploids (∼85%) derived from EAHB. The objectives were to (i) compare the predictive ability of a set of six models with marker, pedigree, and both pedigree and marker information for 15 traits scored in the training population, and select the best genomic prediction model for each trait or a group of traits, (ii) determine the predictive ability of models with a training population grown under two different field management practices (i.e., studying genotype × environment interaction), (iii) determine the predictive ability of the best model for prediction of traits within and across crop cycle 1/mother plants and crop cycle 2/first ratoons/first suckers (i.e., genotype × cycle interaction), (iv) determine the effect of accounting for allele dosage on the predictive ability of the best genomic prediction model for each trait, (v) determine the effect of using genomic prediction models fitted with averaged environment phenotype data and allele dosage SNP (AD‐SNP) markers on the prediction of genotype performance in particular environments and (vi) determine the accuracy of selection based on GEBV relative to phenotypic data within the training population. To achieve these objectives, a training population of 307 banana genotypes consisting of breeding clones and hybrids was phenotyped and genotyped.

## MATERIALS AND METHODS

### Phenotyping

The banana genomic selection training population used in this study and the traits measured were described in detail by Nyine et al. (2017). Briefly, the training population consisted of 307 genotypes that included diploid (11%), triploid (85%), and tetraploid (4%) plants. The core breeding clones (parents) accounted for 12% of the population. The triploid parents were EAHB some of which were crossed with cultivar (cv.) Calcutta 4 to generate tetraploid hybrids, which are used as breeding clones (Supplemental Table S1). The diploid parents consisted of both wild and improved parthenocarpic genotypes. The rest were hybrids from early evaluation trials and advanced clones that had been selected over time during the 20 year of banana breeding by the International Institute of Tropical Agriculture (IITA) and the National Agricultural Research Organization of Uganda. In total, 77 families (cross combinations) of variable sizes were represented in this population. Phenotyping was done at IITA research station located at Sendusu in Namulonge, 0.53° N 32.58° E, 1150 m above sea level with rainfall of about 1200 mm/year split into two rainy seasons, March‐June and September‐December, and an average annual temperature of 22°C.

Two phenotyping fields were established to mimic different agronomic practices that farmers use, thus creating a difference in growth environment. A completely randomized design with three replications per genotype was used to establish the fields. Sword and maiden suckers were used as planting materials with a spacing of 2 × 3 m. In the genomic selection trial one (GS1), 20 kg of manure was applied at planting, but neither mulching, nor nitrogen, phosphorus and potassium (NPK) fertilizer application was done afterward and this was considered a low input field management. The genomic selection trial two (GS2) was planted with 20 kg of manure, then mulched, and NPK fertilizer (25:5:5) was added at a rate of 480 g per plant mat per year, and this was considered a high input field management. In both fields, sucker management was done to maintain a maximum of three plants per mat.

Data were collected on two crop cycles in each field between 2013 and 2016. Fifteen traits were considered for genomic prediction modeling and these were categorized as plant stature, suckering behavior, black leaf streak resistance, fruit bunch, and fruit filling. For plant stature, plant height and girth at 100 cm from soil surface were measured at flowering. The total number of suckers and height of tallest sucker were recorded at flowering of crop cycle 1 and height of tallest sucker at harvest to represent suckering behavior. The number of standing leaves and index of non‐spotted leaves were determined at flowering to characterize black leaf streak resistance. The index of non‐spotted leaves was calculated according to the formula of Craenen (1998) with some modification as reported by Nyine et al. (2017). The fruit bunch traits scored at harvesting included the days to fruit maturity, bunch mass, number of hands, and number of fruits. For fruit filling, fruit length, fruit circumference, fruit diameter, and pulp diameter were measured at harvest. The data were checked for outliers and entry errors prior to use in model fitting. It should be noted that not all traits had full data sets because some genotypes had not completed the second cycle through harvest by the time of these analyses.

### Genotyping

The population was genotyped by sequencing as described by Elshire et al. (2011). The restriction enzyme *Pst*I was used in the genome complexity reduction during sequencing library preparation. Barcodes containing adaptors were ligated to the genomic DNA fragments. Ninety‐six samples were multiplexed and sequenced on a single Illumina lane at the Institute of Genomic Diversity, Cornell University. Each set of 96 samples was run twice to increase the number of reads per *Pst*I tag. Single‐end reads of 100 bp were generated during sequencing. A workflow for the analysis of sequence reads was developed (Supplemental Fig. S1).

Sequence reads were filtered using fastq_quality_filter provided in the module fastx.0.0.13 (‐q 20‐p 90). Sequence reads were subjected to quality control analysis using fastqc provided in module FastQC.0.10.1. Reads from each lane were de‐multiplexed into individual sample reads using fastx_barcode_splitter.pl provided in fastx.0.0.13. The barcodes were trimmed using fastx_trimmer in the module fastx.0.0.13. Any remaining adaptor sequences were removed using fastx_clipper also provided in module fastx.0.0.13. The *Pst*I tag (5’‐TGCAG—–3’) was retained on each sequence read to act as a reference point during read alignment to the reference genome. Reads of the same genotype were merged into one file for downstream analysis. Bowtie2 was used to align reads to the latest publicly available reference banana genome (Martin et al., 2016). Read groups were added to aligned sample reads after which the duplicate reads were marked and removed using picard‐1.100. Indels were realigned and all realigned reads from all samples were merged into one file before SNP calling.

Genome analysis tool kit (GATK) version 2.7.2, UnifiedGenotyper (https://software.broadinstitute.org/gatk/documentation/) was used as the variant caller. First, all genotypes were considered as diploids and as such bi‐allelic SNP (BA‐SNP) were called. Second, the population was split and grouped according to ploidy level. The respective ploidy levels were set during SNP calling. Preliminary filtering of SNP was performed prior to output of variant call file (VCF). The filters used were QD < 2.0, FS > 60.0, MQ < 40 and Haplotypescore > 13.0. Further stringent filtering was done in R (R core team, 2016) where SNP loci with quality score less than 98 and more than 50% of the banana genotypes having missing data were excluded. Concordant SNP loci across all ploidy levels were selected to generate a file with SNP where allele dosage had been accounted for. The remaining missing data were imputed with impute function in R and SNP converted into numerical data for input into genomic prediction models using a custom R‐script. The description of how the script works can be accessed here: http://olomouc.ueb.cas.cz/system/files/users/public/scripts/AlleleDosage_R_function.docx

### Comparison of Genomic Prediction Models and the Effect of Field Management and Crop Cycle on their Performance

Bayesian models accounting for additive genetic effects (Bayesian Ridge Regression [BRR], Bayesian LASSO [BL], BayesA, BayesB and BayesC), and reproducing kernel Hilbert space models with pedigree (P), markers (M), pedigree and markers (PM) accounting for non‐additive genetic effects (RKHS_P, RKHS_M and RKHS_PM) were compared. All models were implemented in R‐package BGLR (Pérez and de los Campos, 2014) using 10807 BA‐SNP markers. Since the training population consisted of many small families and genotypes of different ploidy levels, both phenotype and SNP data were completely randomized in the same order. The aim was to minimize the effect of family structure and ploidy level during cross validation.

The phenotype data used were the average phenotypic observations per genotype per field. These were calculated using the function ‘aggregate’ provided in R‐package plyr. The training population was divided into five groups and each group was used once as the testing (cross validation) set. The predictive ability of the model was determined as the average correlation between the predicted and observed phenotype of the testing sets from five cross validations. Across field management, cross validation was done so that data from one field were used to generate the model using the training set, and the predicted phenotypes of the genotypes in the testing set were correlated to the observed phenotypes in the second field.

For all models, the priors for parameters such as shape, rate, and counts were estimated from the data. However, for BayesB and BayesC models, the prior probability of a marker having a non‐null effect on the phenotype (probIn value) was set at 0.05 and the degrees of freedom were set according to the available phenotype and genotype data. The genetic variance in all models was set at 0.5. For every cross validation, 10,000 iterations were run with a burnIn of 5000 and thin 10.

The fifteen traits mentioned above were predicted with all models to determine the best genomic prediction model for each trait or group of traits. The effect of using models generated with data from low input field management to predict performance of genotypes under high input management and vice versa (G × E effect) was also evaluated.

Next, the effect of crop cycle on trait prediction was evaluated using one of the best identified genomic prediction model. Cross validation across and within crop cycles was done using the 10807 BA‐SNP markers and the average phenotype per crop cycle 1 and crop cycle 2 of each field. Five cross validations were performed without overlap of genotypes between the training and testing set in each round. Only a few traits representing the trait categories were considered because of high correlation of traits within trait categories (Nyine et al., 2017). They included plant girth at 100 cm from soil surface, index of non‐spotted leaves, bunch mass, and fruit circumference. The total number of suckers was not analyzed because this trait was scored only in crop cycle 1.

### Effect of Allele Dosage on Model Performance

The performance of BayesB, BRR, BL, and RKHS_M models fitted with BA‐SNP and AD‐SNP markers was compared for the 15 traits. Predictions based on BA‐SNP markers were used as the baseline for comparison. Equal number of SNP from same loci for both BA‐SNP and AD‐SNP were used. Combined phenotypic data from the two fields for the two crop cycles (environment averaged data) were used to calculate the mean phenotype of each individual genotype. In this cross‐validation strategy, first, genotypes were completely randomized. A five‐fold cross validation was performed using similar priors to determine the predictive ability of the model for the trait. Second, the performance of parents’ model versus progeny's model was compared using BA‐SNP and AD‐SNP. Here, the training set consisted of either only parents (parents’ model), or progeny (progeny's model). Third, the population was divided into three groups consisting of diploids, triploids, and tetraploids. The training set comprised of any two of the ploidy groups while the testing set consisted of genotypes from one ploidy level. Due to differences in population sizes under different ploidy level, we also used only triploids to compare the effect of accounting for allele dosage.

The effect of using averaged environment model was assessed based on AD‐SNP to predict plant girth at 100 cm from soil surface, total number of suckers, index of non‐spotted leaves, bunch mass, and fruit circumference under low and high input fields. The percentage difference in prediction (PDP) between low and high input fields was calculated in reference to the prediction in the low input field management.

To understand the variation and trend of predictive ability across traits, both broad (H^2^) and narrow (h^2^) sense heritabilities were estimated following the methods described by Kruijer et al. (2015). The BA‐SNP markers (10,807) and phenotypic means from each field were used to estimate h^2^ using R‐package heritability while the results from analysis of variance were used to estimate H^2^. Type B genetic correlation was also performed based on phenotypic means from GS1 and GS2 to determine the effect of G × E interaction on the trend of trait prediction across fields (Burdon, 1977).

### The Accuracy of Genomic Prediction within the Training Population

The GEBV obtained from the models fitted with AD‐SNP with best and worst predictive abilities for plant girth, total number of suckers, index of non‐spotted leaves, bunch mass and fruit circumference were used to rank the genotypes. The top 100 genotypes were compared with the best 100 genotypes ranked on the basis of the environment averaged phenotypic data. The number of genotypes out of 100 captured by both GEBV and phenotypic data was reported as the estimated accuracy of genomic prediction within the training population. For this analysis, the best genomic prediction model identified above was used.

## RESULTS

### Genotyping

**Fig. 3:**
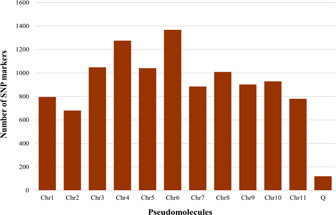
Distribution of filtered SNP markers on 11 pseudomolecules of the double haploid of *M. acuminata* cv. Pahang (Martin et al., 2016). Q represents the unanchored scaffolds.

The discovery of SNP markers from GBS reads for the training population was based on the latest publicly available version of the double haploid *Musa acuminata* cv. Pahang reference genome sequence (Martin et al., 2016). To account for allele dosage in genotypes of different ploidy, a workflow was developed for the analysis of sequence data and GATK, UnifiedGenotyper was used as SNP caller (Supplemental Fig. S1). It produced 52076 BA‐SNP after pre‐filtering. Less than one percent of the loci had multi‐allelic SNP. They were eliminated from the data to avoid potential sequencing artifacts. After further stringent filtering in R (R core team, 2016), 10807 BA‐SNP markers that were polymorphic with a minimum minor allele frequency of 0.01 were retained. These were distributed on 11 pseudomolecules as well as on unanchored scaffold of the banana reference genome (Fig. 3). The percentage of imputed missing genotypes was 16%. Accounting for allele dosage within the ploidy groups (diploids, triploids, and tetraploids) reduced the number of SNP markers to 5574.

### Comparison of Genomic Prediction Models and the Effect of Field Management and Crop Cycle on their Performance

**Fig. 4:**
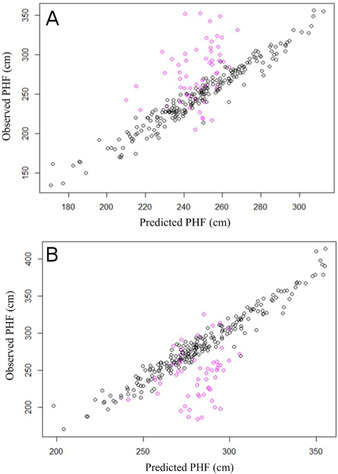
Prediction of plant height at flowering (PHF) using a Bayesian ridge regression model fitted with phenotype data from low input field (A) and high input field (B). Where A, shows underprediction and B, shows overprediction of PHF. The black and magenta circles represent genotypes in the training and testing sets, respectively.

The best genomic prediction model for different traits was selected based on congruity of predictive ability results from cross validation between fields using BA‐SNP markers. The predictive ability of all models varied across traits (Table 1; Supplemental Table S2). For 12 out of 15 traits, genomic prediction models that account for additive genetic effects gave the highest predictions ranging from 0.2 to 0.72. These were the correlations between the predicted and observed phenotypes for the various traits. Reproducing kernel Hilbert space model combining both pedigree and marker information (RKHS_PM) gave the highest predictions ranging from 0.24 to 0.49 for 3 out of 15 traits and these were the days to fruit maturity, height of tallest sucker at flowering and height of tallest sucker at harvesting. BayesB and BayesC models predicted equally well and better than other models for fruit filling and fruit bunch traits. For example, the predictions of all fruit filling traits by both models ranged from 0.65 to 0.72. For plant stature, suckering behavior and black leaf streak resistance traits, BayesB and BayesC models were not the best, but either had the same predictive ability, or were lower by 5 – 13 % in prediction as compared to other models. The trend of prediction starting from the highest to the lowest trait category was fruit filling, fruit bunch, plant stature, black leaf streak resistance, and suckering behavior. In general, genomic prediction models fitted with phenotypic data from GS1 underpredicted the performance of genotypes in GS2, and vice‐versa (Fig. 4), but this did not affect the trend of prediction across traits. Little difference in prediction was observed across all models for traits within the same category.

**Table 1:** Comparison of average correlation (standard errors in parentheses) for five‐fold cross validations between the predicted and observed phenotypes across models fitted with data from either low input (GS1) or high input (GS2) fields and 10807 bi‐allelic SNP markers.

Trait category	Trait	BRR		BayesB		BayesC		RKHS_M		RKHS_PM	
		GS1	GS2	GS1	GS2	GS1	GS2	GS1	GS2	GS1	GS2
Plant stature	Plant height	0.54 (0.06)	0.46 (0.09)	0.54 (0.06)	0.44 (0.09)	0.54 (0.07)	0.45 (0.09)	0.55 (0.06)	0.44 (0.09)	0.54 (0.05)	0.48 (0.07)
	Plant girth	0.60 (0.06)	0.52 (0.05)	0.60 (0.06)	0.52 (0.06)	0.60 (0.06)	0.51 (0.05)	0.60 (0.06)	0.51 (0.06)	0.55 (0.04)	0.50 (0.05)
Suckering behavior	Total number of suckers	0.16 (0.06)	0.17 (0.06)	0.16 (0.06)	0.1(9 (0.06)	0.15 (0.06)	0.19 (0.07)	0.17 (0.06)	0.18 (0.06)	0.16 (0.04)	0.17 (0.07)
	Height of tallest sucker at flowering	0.28 (0.05)	0.18 (0.09)	0.27 (0.05)	0.20 (0.08)	0.26 (0.05)	0.2 (0.08)	0.28 (0.05)	0.19 (0.09)	0.30 (0.06)*	0.24 (0.09)*
	Height of tallest sucker at harvesting	0.27 (0.05)	0.26 (0.07)	0.28 (0.06)	0.24 (0.06)	0.27 (0.06)	0.25 (0.07)	0.26 (0.05)	0.26 (0.06)	0.29 (0.03)*	0.32 (0.07)*
Black leaf streak	Number of standing leaves at flowering	0.36 (0.08)	0.42 (0.08)	0.43 (0.06)	0.40 (0.08)	0.36 (0.08)	0.41 (0.08)	0.37 (0.08)	0.41 (0.08)	0.29 (0.07)	0.34 (0.04)
	Index of non‐spotted leaves	0.35 (0.04)	0.42 (0.06)	0.34 (0.05)	0.43 (0.06)	0.34 (0.05)	0.43 (0.06)	0.35 (0.05)	0.42 (0.06)	0.32 (0.07)	0.36 (0.10)
Fruit bunch	Days to fruit maturity	0.47 (0.07)	0.42 (0.09)	0.47 (0.07)	0.42 (0.09)	0.46 (0.07)	0.42 (0.09)	0.47 (0.07)	0.42 (0.10)	0.49 (0.06)*	0.44 (0.09)*
	Bunch mass	0.63 (0.03)	0.61 (0.03)	0.64 (0.03)*	0.62 (0.03)*	0.64 (0.03)*	0.62 (0.03)*	0.61 (0.03)	0.61 (0.03)	0.52 (0.06)	0.55 (0.04)
	Number of hands	0.60 (0.03)*	0.62 (0.04)*	0.60 (0.02)*	0.62 (0.04)*	0.59 (0.02)	0.62 (0.04)	0.59 (0.03)	0.62 (0.04)	0.48 (0.03)	0.53 (0.02)
	Number of fruits	0.47 (0.03)	0.51 (0.04)	0.47 (0.03)*	0.52 (0.04)*	0.47 (0.02)*	0.52 (0.04)*	0.45 (0.03)	0.52 (0.04)	0.35 (0.04)	0.45 (0.04)
Fruit filling	Fruit length	0.65 (0.04)	0.64 (0.02)	0.67 (0.04)*	0.65 (0.02)*	0.67 (0.03)*	0.65 (0.02)*	0.64 (0.04)	0.64 (0.02)	0.59 (0.07)	0.59 (0.02)
	Fruit circumference	0.67 (0.02)	0.66 (0.01)	0.70 (0.01)*	0.69 (0.01)*	0.70 (0.01)*	0.69 (0.01)*	0.65 (0.02)	0.66 (0.01)	0.57 (0.05)	0.60 (0.02)
	Fruit diameter	0.67 (0.01)	0.63 (0.05)	0.70 (0.01)*	0.71 (0.02)*	0.70 (0.01)*	0.71 (0.02)*	0.65 (0.02)	0.67 (0.03)	0.57 (0.04)	0.59 (0.02)
	Pulp diameter	0.67 (0.02)	0.68 (0.04)	0.70 (0.01)*	0.72 (0.03)*	0.70 (0.01)*	0.72 (0.03)*	0.65 (0.02)	0.67 (0.04)	0.57 (0.04)	0.60 (0.03)

* Highest predictive value observed in both GS1 and GS2 for a trait using same model type. The values under GS1 column are the correlations between predicted and observed phenotype (predictive ability) in GS2 when GS1 data were used to fit the model and vice versa for GS2 column.

The performance of RKHS model fitted with marker data (RKHS_M) was comparable to BRR, BL, and BayesA models fitted with marker data. RKHS model fitted with pedigree information alone (RKHS_P) had the least predictive ability that ranged from 0.12 to 0.5 (Supplemental Table S2). There was a 4 to 29% loss in predictive ability (LIP) of most traits when marker and pedigree information were combined in the RKHS_PM model. However, the same model gave a 4 to 21% gain in prediction for plant height, height of tallest sucker at flowering, height of tallest sucker at harvesting and days to fruit maturity.

The effect of crop cycle on trait prediction was tested with BayesB model using BA‐SNP markers, because this model either out‐performed other models, or performed equally well as noted in Table 1; Supplemental Table S2. The cross‐validation strategies used were (a) within crop cycle cross validation for which both the training and testing sets were from the same crop cycle and (b) across crop cycle cross validation where the training and testing sets were selected from different crop cycles within the same field. The predictive ability of BayesB model fitted with crop cycle 1, or crop cycle 2 data in both low input and high input fields yielded mixed results when within and across crop cycle cross validations were performed for different traits (Table 2). Predictive ability of the model for fruit circumference and bunch mass ranged from 0.58 to 0.73, while for plant girth and index of non‐spotted leaves ranged from 0.39 to 0.61 and 0.26 to 0.44, respectively, in both fields and crop cycles. Less than 2% variation in prediction across and within crop cycles was observed in both bunch mass and fruit circumference. The highest difference of 20% in prediction across (0.28) and within (0.35) crop cycle was recorded in GS2 for index of non‐spotted leaves when crop cycle 2 data were used to fit the model.

**Table 2:** Average predictive ability (standard errors in parentheses) of BayesB model fitted with either crop cycle 1, or crop cycle 2 phenotype data from low (GS1) and high (GS2) input field management using bi‐allelic SNP markers to predict traits across and within crop cycles.

		Low input field management (GS1)				High input field management (GS2)			
		Cycle 1 model		Cycle 2 model		Cycle 1 model		Cycle 2 model	
Trait category	Trait	Across	Within	Across	Within	Across	Within	Across	Within
Plant stature	Plant girth	0.39 (0.04)	0.55 (0.03)	0.51 (0.02)	0.44 (0.05)	0.54 (0.02)	0.59 (0.02)	0.61 (0.02)	0.57 (0.02)
Black leaf streak	Index of non‐spotted leaves	0.42 (0.06)	0.44 (0.03)	0.40 (0.04)	0.41 (0.03)	0.30 (0.08)	0.26 (0.04)	0.28 (0.05)	0.35 (0.05)
Fruit bunch	Bunch mass	0.58 (0.03)	0.60 (0.04)	0.60 (0.06)	0.59 (0.03)	0.63 (0.02)	0.65 (0.03)	0.65 (0.02)	0.62 (0.03)
Fruit filling	Fruit circumference	0.72 (0.02)	0.71 (0.03)	0.72 (0.04)	0.72 (0.02)	0.73 (0.02)	0.73 (0.03)	0.71 (0.02)	0.72 (0.02)

### Effect of Allele Dosage

The effect of AD‐SNP on predictive ability of the best genomic prediction models was evaluated for 15 traits in comparison to predictions based on BA‐SNP markers. For both BA‐SNP and AD‐SNP, 5574 SNP markers from the same loci and combined phenotypic data from the two fields for the two crop cycles (environment averaged data) were used to fit the models. First, genotypes were completely randomized to minimize the effect of family structure and ploidy. Second, the training set consisted of either only parents (parents’ model), or progeny (progeny's model). Third, the population was divided into diploids, triploids, and tetraploids. The training set comprised of any two of the ploidy groups while the testing set consisted of genotypes from one ploidy level. Lastly, only triploids were considered during cross validation since 85% of genotypes in the training population were triploids. The aim was to understand what traits and which ploidy level were mostly affected by allele dosage when implementing genomic predictions.

The results of the comparison of the effect of allele dosage on performance of BayesB, BRR, BL, and RKHS_M models are summarized in Table 3. When AD‐SNP were used to fit the models, predictive ability of all models was trait dependent, but generally reduced by 15% on average as compared to the traditional BA‐SNP markers. When only triploids were considered during the cross validation, predictive ability for fruit circumference fell by 10% from 0.76 to 0.68, while for bunch mass it decreased by 5% from 0.62 to 0.59. The highest loss in prediction (PLP) of 24 to 44% was observed in suckering behavior traits when AD‐SNP markers were used to fit model using genotypes from all ploidy levels. However, the trend of prediction within and across trait categories did not change by accounting for allele dosage. Fruit filling traits were the best predicted with the highest predictive ability of 0.68 for pulp diameter. BayesB model maintained its superior prediction accuracy over other models, especially for fruit filling and fruit bunch traits.

**Table 3:** Effect of accounting for allele dosage on the predictive ability of genomic prediction models using environment averaged phenotype data.

		Bi‐allelic SNP				Allele dosage SNP			
Trait category	Trait	BRR	BayesB	BL	RKHS_M	BRR	BayesB	BL	RKHS_M
Plant stature	Plant height	0.54 (0.03)†	0.53 (0.02)	0.52 (0.03)	0.53 (0.03)	0.46 (0.07)	0.45 (0.06)	0.44 (0.07)	0.45 (0.07)
	Plant girth	0.53 (0.04)	0.53 (0.03)	0.52 (0.04)	0.52 (0.04)	0.48 (0.04)	0.47 (0.04)	0.47 (0.04)	0.48 (0.04)
Suckering behavior	Total number of suckers	0.32 (0.06)	0.29 (0.06)	0.33 (0.05)	0.31 (0.06)	0.21 (0.05)	0.16 (0.05)	0.21 (0.05)	0.21 (0.05)
	Height of tallest sucker at flowering	0.37 (0.04)	0.34 (0.04)	0.37 (0.04)	0.38 (0.04)	0.27 (0.06)	0.26 (0.05)	0.27 (0.05)	0.28 (0.05)
	Height of tallest sucker at harvesting	0.35 (0.04)	0.33 (0.03)	0.34 (0.04)	0.35 (0.04)	0.24 (0.03)	0.23 (0.03)	0.23 (0.03)	0.25 (0.03)
Black leaf streak	Number of standing leaves at flowering	0.49 (0.05)	0.48 (0.05)	0.48 (0.05)	0.48 (0.05)	0.48 (0.06)	0.48 (0.06)	0.48 (0.06)	0.49 (0.06)
	Index of non‐spotted leaves	0.58 (0.03)	0.59 (0.03)	0.58 (0.03)	0.58 (0.03)	0.53 (0.03)	0.52 (0.03)	0.53 (0.04)	0.53 (0.03)
Fruit bunch	Days to fruit maturity	0.53 (0.05)	0.54 (0.06)	0.53 (0.06)	0.53 (0.06)	0.44 (0.05)	0.43 (0.05)	0.44 (0.05)	0.44 (0.05)
	Bunch mass	0.61 (0.05)	0.62 (0.04)	0.61 (0.05)	0.61 (0.04)	0.54 (0.03)	0.56 (0.03)	0.54 (0.03)	0.54 (0.02)
	Number of hands	0.63 (0.04)	0.62 (0.04)	0.62 (0.04)	0.63 (0.04)	0.56 (0.03)	0.56 (0.03)	0.56 (0.03)	0.56 (0.03)
	Number of fruits	0.49 (0.04)	0.49 (0.04)	0.48 (0.04)	0.50 (0.04)	0.43 (0.03)	0.42 (0.04)	0.42 (0.03)	0.43 (0.04)
Fruit filling	Fruit length	0.69 (0.02)	0.70 (0.02)	0.69 (0.03)	0.69 (0.02)	0.60 (0.03)	0.64 (0.02)	0.60 (0.02)	0.59 (0.03)
	Fruit circumference	0.67 (0.03)	0.75 (0.02)	0.68 (0.03)	0.66 (0.03)	0.59 (0.03)	0.66 (0.03)	0.60 (0.03)	0.59 (0.03)
	Fruit diameter	0.67 (0.03)	0.75 (0.02)	0.68 (0.03)	0.66 (0.03)	0.60 (0.03)	0.67 (0.03)	0.62 (0.02)	0.60 (0.02)
	Pulp diameter	0.68 (0.03)	0.75 (0.03)	0.69 (0.03)	0.67 (0.03)	0.61 (0.03)	0.68 (0.03)	0.63 (0.03)	0.61 (0.02)

† The values in parentheses are the standard errors of predictive ability.

Although the number of SNP markers used in this prediction was reduced to 5574 because we wanted to eliminate the bias in predictions due to variable number and location of BA‐SNP and AD‐SNP, the environment (field management) averaged models with BA‐SNP markers gave higher predictions than those obtained with across field cross validation with 10,807 SNP markers for all traits. The highest predictive ability recorded was 0.75 for fruit filling traits with the BayesB model (Table 3).

When only parental data were used to fit BayesB model (parents’ model), the predictive ability of traits within the progeny ranged from 0.13 to 0.59 for BA‐SNP and from ‐0.15 to 0.33 for AD‐SNP (Supplemental Table S3). The LIP due to accounting for allele dosage was 63% on average (36–179%). Similarly, when progeny data were used to fit BayesB model (progeny's model), the predictive ability of traits within parents ranged from 0.39 to 0.86 with BA‐SNP and from ‐0.03 to 0.77 with AD‐SNP markers. The LIP due to accounting for allele dosage was 35% on average (1.5–107%). The highest predictive value obtained with BayesB model fitted with BA‐SNP was 0.86 for number of hands. This prediction dropped by nearly 50% (0.48) when AD‐SNP markers were used. Prediction accuracy of the same trait in progeny using parents’ model was 0.45 with BA‐SNP and 0.03 with AD‐SNP markers. The prediction of bunch mass in the progeny using a parents’ model with AD‐SNP was 0.17 while the prediction of the same trait in parents using a progeny's model reduced to 0.08.

Since allele dosage varies with ploidy level, cross validation across ploidy levels was performed. Genotypes from two ploidy levels were used to train the model and only genotypes of same ploidy level were included in the testing set during cross validation. Accounting for allele dosage positively increased the predictive ability of all fruit filling traits in tetraploids with BayesB model, but the results from other trait categories varied greatly (Supplemental Table S4). For example, prediction of pulp diameter increased from ‐0.39 to 0.60, fruit diameter increased from ‐0.45 to 0.53 and fruit circumference increased from ‐0.15 to 0.35. BayesB model fitted with triploid and tetraploid data, and BA‐SNP gave the predictions ranging from 0.32 to 0.86 for traits among diploids. Tetraploids and diploids were the least represented in the training population (47 out of 307 genotypes, or 15%) and of which the majority were parents. When their data were used to fit the model to predict traits in triploids the prediction varied from 0.20 to 0.54 and from ‐0.06 to 0.11 with BA‐SNP and AD‐SNP, respectively.

When BayesB model was fitted with the environment averaged data (including all ploidy levels) and AD‐SNP to predict the traits under low and high input fields, there was a 2 to 8% increase in predictive ability under high input field relative to low input field for plant girth, bunch mass and fruit circumference (Table 4). However, for total number of suckers and index of non‐spotted leaves, the predictions reduced by 47 and 15%, respectively.

**Table 4:** Performance of BayesB model fitted with average phenotype data for all fields (environments) and AD‐SNP markers for predictions of five traits representing the trait categories within low and high input fields.

Trait category	Trait	Low input field (GS1)	High input field (GS2)	Percentage loss in prediction (PDP)
Plant stature	Plant girth	0.48 (0.07) †	0.52 (0.08)	8.3
Suckering behavior	Total number of suckers	0.15 (0.05)	0.08 (0.05)	−46.7
Black leaf streak	Index of non‐spotted leaves	0.39 (0.06)	0.33 (0.05)	‐15.4
Fruit bunch	Bunch mass	0.56 (0.05)	0.57 (0.05)	1.8
Fruit filling	Fruit circumference	0.66 (0.01)	0.69 (0.03)	4.5

† The values in parentheses are the standard errors of predictive ability, PDP is percentage difference in prediction.

Estimated H^2^ and h^2^ had positive relationship with predictive ability. However, h^2^ varied across fields with some traits having higher h^2^ than H^2^ (Table 5). A similar trend was observed between Type B genetic correlation and predictive ability. The correlation varied between 0.71 and 0.9 for fruit bunch, fruit filling and plant stature traits. The lowest correlation was recorded on the index of non‐spotted leaves (Table 5).

**Table 5:** Estimated broad (H^2^), narrow (h^2^) sense heritability within low (h^2^_GS1) and high (h^2^_GS2) input fields and type B genetic correlation (*r*) between GS1 and GS1.

Trait category	Trait	H^2^	h^2^_GS1	h^2^_GS2	r GS1/GS2 (type B)
Plant stature	Plant height	0.89	0.99	0.93	0.79
	Plant girth	0.90	0.93	0.91	0.83
Suckering behavior	Total number of suckers	0.80	0.45	0.36	0.49
	Height of tallest sucker at flowering	0.82	0.70	0.93	0.56
	Height of tallest sucker at harvesting	0.86	0.41	0.84	0.47
Black leaf streak	Number of standing leaves at flowering	0.83	0.63	0.81	0.54
	Index of non‐spotted leaves	0.72	0.72	0.63	0.38
Fruit bunch	Days to fruit maturity	0.89	0.65	0.85	0.71
	Bunch mass	0.94	0.96	0.95	0.86
	Number of hands	0.93	0.91	0.91	0.81
	Number of fruits	0.89	0.97	0.94	0.74
Fruit filling	Fruit length	0.96	0.97	0.98	0.84
	Fruit circumference	0.97	0.94	0.96	0.87
	Fruit diameter	0.97	0.93	0.99	0.89
	Pulp diameter	0.97	0.93	0.92	0.90

### The Accuracy of Genomic Prediction within the Training Population

**Fig. 5:**
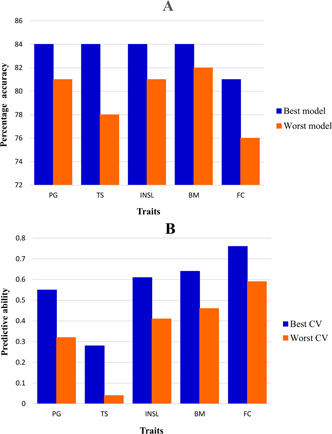
Accuracy of genomic prediction in the training population. (A) Percentage of genotypes selected by both GEBV and phenotypic data within the first best ranked 100 genotypes. (B) Correlations of the best and worst BayesB models used to generate GEBV. Where, PG is plant girth at 100 cm from soil surface, TS is total number of suckers, INSL is index of non‐spotted leaves, BM is bunch mass, FC is fruit circumference and CV is cross validation.

The first 100 genotypes with the highest GEBV and the first 100 genotypes with the highest environment averaged phenotypic data were compared (Fig. 5). The GEBV used were obtained from BayesB model with best and worst predictive abilities based on AD‐SNP markers. The number of genotypes out of 100 captured by both GEBV and phenotypic data was reported as the estimated accuracy of genomic prediction within the training population for the trait. The accuracy of prediction ranged from 76 to 84% for all the traits whereas the prediction values ranged from 0.04 to 0.76. Models that gave high predictive ability values had also the highest prediction accuracy.

## DISCUSSION

### Genotyping

Genomic selection as a form of marker assisted selection has been investigated in a range of plant species including, for example, maize and wheat (Heffner et al., 2011; Crossa et al., 2014; Crossa et al., 2016; Pérez‐Rodríguez et al., 2017), white spruce (Beaulieu et al., 2014), sugar beet (Würschum et al., 2013), apples (Kumar et al., 2012), strawberries (Gezan et al., 2017), and rice (Onogi et al., 2016). In these experiments, genotypes of same ploidy level constituted the training population. The present study on banana is unique in this respect as three ploidy levels were represented in the training population. Within the three ploidy levels, both parents and progeny were represented in varying proportions. The hybrids in the training population arose from 77 cross combinations, mainly involving crosses between tetraploids and diploids (Nyine et al., 2017). Innovative approaches in SNP calling, including custom R‐script had to be adopted for such an unconventional population (Supplemental Fig. S1). The script removes loci with monomorphic SNP, eliminates loci with more than two alternative SNP alleles, and converts the SNP file into a numerical format while accounting for allele dosage, and it can be customized to any polyploid plant species. Loci with multi‐allelic SNP were eliminated because GBS is a low coverage sequencing technology. This makes it hard to differentiate true rare SNP from sequence artifacts especially when the population is small and the species is clonally propagated due to lower rate of multiple mutations at the same locus. Bowtie2 was used as the sequence alignment tool while GATK, UnifiedGenotyper was the variant caller. However, as indicated by Clevenger et al. (2015), optimal alignment programs and variant callers may vary among species.

GATK (https://software.broadinstitute.org/gatk/documentation/) in particular is useful when handling polyploid species. It allows setting the ploidy level and reduces false positive SNP calls arising from frameshifts by running INDEL realignment step (Clevenger et al., 2015). When Picard tools (https://sourceforge.net/projects/picard/files/picard‐tools/1.100/) are used prior to SNP calling, normalization of sequence reads is possible by marking and removing duplicate reads. This allows regions with low reads coverage, but carrying SNP of interest to be included in the genotype data. Picard tools also allow merging of aligned sample reads by addition of read groups, which help in separating genotypes after SNP calling.

### What is the Best Genomic Prediction Model for Each Trait or Group of Traits?

Different genomic prediction models were compared in this work in terms of their predictive ability, or accuracy for different traits as noted in Table 1 and Supplemental Table S2. We compared the performance of models that account for additive genetic effects and those that account for non‐additive genetic effects. A good performance of models that account for additive genetic effects suggested that a large proportion of phenotypic variation observed in the training population was due to additive genetic effects. Indeed, traits with high narrow sense heritability (h^2^) had higher predictive values. A similar observation was made by Luan et al. (2009). They reported a strong relationship between prediction accuracy and trait heritability in Norwegian red cattle. Differences in h^2^ between GS1 and GS2, and H^2^ were attributed to bias in residual error variance. Using phenotypic means reduces error variance leading to over estimation of h^2^ as compared to replicated phenotypic data used in estimating H^2^. Usually, proper estimation of heritability requires balanced phenotypic data (Piepho and MÖhring, 2007). However, it is hard to get balanced data for bananas because growth is not synchronized between plants as well as data collection, which causes high variation between genotypes and replicates in the same environment. Generally, H^2^ is specific to a given population at a given location and period, but depending on the genetic architecture of the trait correlations might be observed across populations. For example, our H^2^ results are comparable to those summarized by James et al. (2012) from various publications on bananas and plantains.

Additive genetic effect models BayesB and BayesC performed better than or equally well as other models. These models perform both shrinkage and variable selection on markers to include in the model (Desta and Ortiz, 2014). The prior probability of a marker having a non‐null effect (π) was set at 0.05 in both models because it gave the highest predictive ability values as compared to higher prior settings. It is likely that the same markers were selected and included in both models thus yielding closely related results.

Our results agree with other studies, which indicate that models that perform specific shrinkage and variable selection give better predictive ability values. For example, Crossa et al. (2010) showed that a BL model that shares some characteristics with BayesB outperformed BLUP, which assumes equal variance for each marker. Similarly, Clark et al. (2011) reported the superiority of BayesB model over genomic BLUP. They argued that the superiority was highly dependent on the presence of large QTL effects. In relation to this argument, it is likely that even in banana, fruit filling traits could be controlled by large effect QTL that were selected by BayesB model in all cross‐validations. However, this remains to be proved by QTL mapping and genome‐wide association studies that are out of the scope of this study. Tagging of loci controlling fruit filling with DNA markers and selecting for favorable alleles should also be considered. Fruit filling is a bunch mass component that reflects the sink capacity of a fruit bunch. It was treated separately from other bunch mass components to better describe the proportion of edible part of the fruit. Variation in performance of models that perform shrinkage and variable selection has also been reported. For example, in loblolly pine, BayesCπ (Habier et al., 2011) and BayesA had better prediction of fusiform rust disease‐resistance traits than BL (Resende et al., 2012)

The predictive ability of all models varied across traits. Similar predictive values for traits within the same category confirmed the findings of Nyine et al. (2017) who reported a high correlation between these traits and recommended that only traits easier to phenotype should be considered for genomic predictions. The difference in model performance between trait categories suggests that variation in trait architecture, number of QTL controlling the trait and linkage disequilibrium between markers and QTL influence the performance of the models (Clark et al., 2011).

The RKHS_PM model, which accounts for non‐additive genetic effects yielded mixed prediction results. While some traits had a slight increase in prediction, a majority showed loss in predictive ability (Table 1; Supplemental Table S2). Previous studies (Crossa et al., 2010) indicated minor improvement in trait prediction in wheat and maize when marker and pedigree information were included in the model. However, Pérez‐Rodríguez et al. (2017) reported better prediction with RKHS_P for wheat lines in international environments. The contradictions could be attributed to the training population structure. Our training population consisted of 77 subfamilies (cross combinations) of varying sizes with diverse pedigree background (Nyine et al., 2017). This suggests that when the population consists of many subfamilies, the relationship by pedigree becomes less important. This is reflected by the poor performance of RKHS_P model, which gave the least prediction accuracy for all traits (Supplemental Table S2). A similar trend was observed by Beaulieu et al. (2014). Hence, the estimates of allele distribution within such a population is better performed with marker data, while addition of pedigree information distorts the relationship between the genotypes. Zhong et al. (2009) also highlighted that knowledge of pedigree is less informative in populations where the average genetic relationship is low and homogeneity is high.

### What is the Effect of G × E on Model Predictions?

We used a very conservative approach in determining the best genomic prediction model by carrying out across field (environment) cross validations. The purpose was to understand the effect of genotype by field management (G × E) interaction on the model performance. Nyine et al. (2017) performed analysis of variance on the same population and reported a variation in G × E interaction across different traits. However, type B genetic correlations (Table 5) were high for traits related to fruit bunch and fruit filling, which explains why they had high predictive ability values across all cross‐validation strategies. When Burdon (1977) proposed the use of type B genetic correlation, he noted that in the analysis of variance, any genetic expression variation between environments can lead to statistical interaction that is not necessarily a true interaction characterized by a change in ranking of genotypes between different environments. The results showed that models fitted with GS1 phenotype data underpredicted the phenotypic expression of genotypes in GS2 while the models fitted with GS2 phenotype data overpredicted genotypes in GS1 (Fig. 4). However, the trend of prediction did not change (Table 1). A similar approach was used by Ly et al. (2013), who observed that across environment cross validations resulted into lower prediction accuracies. However, our prediction values were substantially higher as compared to those reported in other crops.

Trait overprediction in GS1 with models fitted with GS2 data and vice versa indicated a variation in genotype response to environment that influenced the training population trait mean, estimated marker effect and the predictive ability of the genomic prediction models (Crossa et al., 2016). The high correlation between the two fields shows that it is possible to use phenotype data from any of the field management conditions to predict genotypes that have the potential to perform well in other field management conditions. However, the predicted and the actual observed phenotype may differ for a single genotype. For example, plants that had poor fruit filling characteristics under low input field management did not fill under high input field management, as well. However, for genotypes that fill their fruits, there was an increase in fruit size depending on the amount of available nutrients and soil moisture in the field. A similar trend was reported in maize flowering where QTL were consistent across environments and less affected by environment interaction (Buckler et al., 2009). This means that genomic prediction models could be used in ‘negative selection’ to discriminate the poor fruit filling hybrids from those with potential of fruit filling at an early stage.

In banana breeding, most triploid hybrids are sterile. The application of genomic prediction in its strict sense of selecting best parents for further crossing (Meuwissen et al., 2001; Gorddard and Hayes, 2007) may not be realistic, unless the focus is only on diploid and tetraploid improvement. Since the prediction models give both GEBV and predicted phenotype (Pérez and de los Campos, 2014), these two parameters can be used to eliminate triploid hybrids that are likely to be of no value. Crossa et al. (2014) also proposed that another application of genomic prediction was to predict the genetic values of individuals for potential release as cultivars. Therefore, if the prediction accuracy remains high during the breeding phase, then breeders could save time, space, and money by excluding 90% of hybrids from phenotyping (Fig. 2). To achieve this, breeders have to set priority order of traits, which could serve as the ‘selection index’ for promising candidate cultivars (i.e., within triploids hybrids) and future parental clones (within diploid and tetraploid hybrids). Also, family based selection should be done to reduce future inbreeding and maximize genetic diversity to ensure increase in genetic gain (Jannink et al., 2010).

Although crop cycle was shown to influence variation in fruit filling, fruit bunch and plant stature, and no effect on black leaf streak resistance traits (Nyine et al., 2017), the predictions within and across crop cycle 1 and crop cycle 2 did not vary much for fruit filling and fruit bunch traits. This is because fruit filling and fruit bunch traits increase in crop cycle 2 relative to crop cycle 1 (Tushemereirwe et al., 2015). However, for black leaf streak resistance, resistant hybrids remain resistant across crop cycles and field management. Variation may be observed among susceptible hybrids depending on the spore density in the field (Tushemereirwe, 1996). Disease expression also depends on vigor of the plant due to available nutrients, seasonal changes and relative humidity in the field (Tushemereirwe, 1996). This probably explains the variation observed in the prediction within and across crop cycle for the index of non‐spotted leaves.

In bananas, suckering behavior traits had the lowest prediction accuracy. One possible explanation is the low heritability and poor representation of markers linked to the QTL controlling these traits. Second, scoring total number of suckers at crop cycle 1 from a trial established with suckers, seems to result in biased phenotype data. Two types of suckers are used as planting materials, the sword and maiden suckers. Most maiden suckers are much closer to flowering than sword suckers (Ortiz and Vuylsteke, 1994) and tend to direct most of resources toward the initiation of the inflorescence, and less to the development of lateral buds (future suckers). On the contrary, sword suckers commit most of their resources to lateral bud development. Hence, when a field is established with suckers, the variation in physiological age of suckers likely impacts sucker emergence that causes bias in total number of suckers produced by a genotype at first crop cycle.

When environment averaged models were used to predict the performance of genotypes in a particular environment, the predictions were high (0.75 for fruit filling traits) despite the lower number of SNP markers (Table 3). This indicated that incorporation of data from many environments could make the models more robust (Burgueño et al., 2012). As discussed by Burgueño et al. (2012), breeders either evaluate new breeding lines so that they can select the best to advance, or evaluate the performance stability of new, or old lines in a new environment. In each of these cases, the model should be robust enough to give accurate predictions in the respective environments (Pérez‐Rodríguez et al., 2017). Hence, using data from multi‐environment trials and crop cycles to fit the model has the advantage of incorporating information due to genetic relationship and the interaction between genotype and environment (Crossa et al., 2014).

Traits that are stable across environments are much easier to predict using data from one environment. However, if there is a proportional change (collinearity) in the trait expression within an environment across genotypes, then selection based on predictions is likely to be efficient (Burgueño et al., 2012). Plant environments vary and may refer to geographical locations with different weather and climatic conditions, difference in seasons within a same location and difference in soil conditions based on the different agronomic practices used. As perennial plants, bananas suffer the consequences of nutrient deficiency and soil moisture variation across seasons and locations depending on field management practices (Ndabamenye et al., 2012; Taulya, 2015). These factors influence phenotypic expression of traits and are likely to affect the predictive ability of prediction models. Although we considered field management and crop cycle as the major environment co‐variables, phenotyping of the current training population in a different geographical location is ongoing. Once the data are available, they will be used to update the models to the benefit of the breeding program.

### Bi‐Allelic SNP vs. Allele Dosage SNP

Whereas many factors have been reported to influence the accuracy of genomic predictions (Crossa et al., 2014), our results showed that allele dosage was another important factor to consider when conducting predictions in multi‐ploidy populations (Supplemental Table S4). The loss in predictive ability of the models fitted with AD‐SNP relative to those fitted with BA‐SNP could be attributed to variation in minor allele frequency across loci, a key factor for determining SNP effects on the traits and the allopolyploid nature of the training population. The negative correlations observed from across ploidy cross validation indicated a weak relationship between the training and testing sets (Crossa et al., 2016). Clearly, not all traits were affected equally by allele dosage (Supplemental Table S4). The effect of allele dosage becomes more important as the ploidy level increases. This suggests that additive genetic effects vary across traits. It is likely that the effect of deleterious recessive alleles is masked by the dominant alleles and the more copies of masking alleles the better the effect (Gu et al., 2003). However, for traits controlled by exclusively recessive alleles, the effect of allele dosage may be different. In cassava, a large proportion of deleterious alleles arising from mutations have not been eliminated by breeding due to limited recombination, but the maintenance of cassava yield through breeding has been attributed to masking of most damaging mutations (Ramu et al., 2017).

Predictions within multi‐family population was shown by Heffner et al. (2011) to be accurate and cost effective. It is likely that genomic prediction models trained only on diploid segregating populations would be less efficient in prediction of traits among triploid banana hybrids, yet promising candidate cultivars are selected in this ploidy level. Second, allele dosage could be accounted for in the marker data especially when predicting fruit filling in tetraploids although use of models that assume diploid state of all genotypes still performed better in many cross‐validation strategies.

To ensure that good hybrids are not left out, selection based on GEBV should be done with prior knowledge of ploidy level in multi‐ploidy populations. Bunch mass and general phenology in bananas tend to increase with increase in ploidy level although in banana hybrids, the trend is not always uniform due to positive and negative heterosis (Tenkouano, 2000). Since banana breeding involves crossing parents of different ploidy levels, prediction of hybrid performance based on parental phenotype data is less accurate due to heterosis. That is why the parents’ model prediction accuracies were low. Although we did not measure heterosis in this study, the results of selection differential and response to selection reported by Nyine et al. (2017) show that it exists in this training population.

When the progeny's model was used to predict the parental traits, the predictions were appreciably high (Supplemental Table S3). This indicated that a large size of the training set relative to the testing set improves prediction (Jannink et al., 2010; Clark et al., 2011; Crossa et al., 2014). The lesson learned is that in bananas, when the training population is made up of many diverse hybrids, the segregation of parental alleles is observed. Most of the additive genetic effects, heterosis, dominance, and epistasis that control the phenotype are captured in the model when all these phenotypic variants are available (Lorenz et al., 2011). These results suggest that for plant species with small effective breeding population sizes like banana that show heterosis, increasing the number of progeny from several parental crosses in the training population could improve the predictive ability of the models for future hybrids as compared to using only parental clones.

### The Accuracy of Genomic Prediction

The prediction accuracy within the training population based on GEBV was above 75% even with models that had low predictive abilities. The accuracy of genomic prediction model is determined by the correlation between GEBV and the observed phenotype, or the correlation between predicted phenotype and observed phenotype (Jannink et al., 2010; Lorenz et al., 2011). This shows the proportion of genetic variance explained by marker data. It is therefore not surprising that even with low correlations, the accuracy of prediction can be high. Beaulieu et al. (2014) reported that with GEBV accuracies between 0.33 and 0.44, they were able to achieve 90% of traditionally estimated breeding values during validation. Similarly, Heffner et al. (2011) reported a 95% prediction accuracy of genomic prediction compared to phenotypic selection in a multi‐family wheat population even when the predictive values ranged from 0.22 to 0.76.

The true accuracy is estimated at the validation stage using the validation population. It depends on the size of the training population, heritability of the trait and the estimated number of effects (Lorenz et al., 2011). Sometimes, it is not possible to explain all the genetic variance due to missing marker data, or failure to capture other QTL affecting the trait. This is further confounded by uncontrolled environmental variable (Buckler et al., 2009; Burgueño et al., 2012). That is why genomic selection is considered less accurate than phenotypic selection but its power lies in increased selection intensity within a much shorter time hence increasing the genetic gain per unit time and cost (Desta and Ortiz, 2014; Lorenz et al., 2011). Our results suggest that even with low predictive values, the accuracy of prediction within the training population was high. It remains to be verified at the validation stage if the accuracy remains high. Given the long selection cycle observed in banana as depicted in Fig. 2, prediction accuracies above 70% could result in accelerated selection efficiency at reduced cost as compared to phenotypic selection.

### Conclusion and Practical Implications

Polyploid breeding programs ought to use genomic prediction models that have been fitted with data from genotypes of all ploidy levels otherwise genomic selection will face similar limitations as other MAS techniques, which focus on bi‐parental populations for QTL and marker discovery. Fruit filling and fruit bunch traits had the highest predictive ability hence, could be targeted for early selection of hybrids. Accounting for allele dosage in SNP markers (AD‐SNP) reduced predictive ability of the models relative to traditional bi‐allelic SNP (BA‐SNP). Unlike autopolyploid, allele dosage seems to have less influence on genomic prediction in allopolyploid populations. However, if ploidy specific prediction models are required, the R script reported could be used to generate AD‐SNP. The heritability of traits estimated in this training population were high and positively correlated with the predictive ability. The results demonstrate that genomic prediction in multi‐ploidy population is possible and the prediction accuracy can be improved by using models based on data from many different environments.

To generate prediction models for each ploidy level is expensive in the initial stages of genomic selection, but as the training population keeps growing it becomes possible. To minimize costs, the current models based on multi‐ploidy population should be validated and used with the following recommendations: (i) unlike other breeding programs where genomic prediction is used entirely for prediction of best parents for further crossing, in banana, selection among triploids should aim at identifying promising candidate cultivars because a majority of them are sterile and breeding clones should be selected from diploids and tetraploids, (ii) ‘selection index’ is required for efficient selection of new hybrids, i.e., the priority order of traits should be set for promising cultivars and breeding clones, (iii) family‐based (cross combination) selection should be considered to avoid reducing genetic diversity, (iv) the lowest GEBV should be targeted for plant height, or else a ratio of plant height to plant girth at 100 cm from soil surface should be used. In the light of genomic selection, a potential area of research would be to investigate the level of fertility in triploid banana hybrids so that they are also selected as parents. This will allow ‘progressive’ breeding to be practiced in banana for faster genetic gain since some traits are already fixed in the triploids.

### Supplemental Material

Supplemental Table S1: List of banana genotypes used in genomic predictions.

Supplemental Table S2: Comparison of average correlation for five‐fold cross validations between the predicted and observed phenotypes across all models fitted with data from either low input (GS1) or high input (GS2) fields and 10807 bi‐allelic SNP markers.

Supplemental Table S3: Comparison of predictive ability of BayesB model fitted with parents’ data and progeny's data using bi‐allelic and allele dosage SNP markers.

Supplemental Table S4: Effect of ploidy level and allele dosage on the predictive ability of BayesB model fitted with environment averaged phenotype data.

Supplemental Fig. S1: Workflow used to analyze the genotyping by sequencing (GBS) reads to generate SNP marker data used in genomic predictions.

### Conflict of Interest Disclosure

The authors declare that there is no conflict of interest.

### Funding

This work was supported by donors through their contributions to the CGIAR Fund (http://www.cgiar.org/our‐funders/), and in particular to the CGIAR Research Program, Roots, Tubers and Bananas. Additional funding was obtained from Bill and Melinda Gates foundation, investment ID: OPP1093845, under the project: Improvement of banana for smallholder farmers in the great lakes region of Africa. Part of this work was supported by the Czech Ministry of Education Youth and Sports (award LO1204 from the National Program of Sustainability I and INGO II award LG15017) while the computing was supported by the Czech National Grid Infrastructure MetaCentrum (grant No. LM2010005 under the program, Projects of Large Infrastructure for Research, Development, and Innovations).

## Supporting information

Supplemental Information
Supplemental File 1
