# Supplementary material for: Genomic Prediction in a Multiploid Crop: Genotype by Environment Interaction and Allele Dosage Effects on Predictive Ability in Banana
Source: Plant Genome. 2018 Jul 1;11(2):170090. doi: 10.3835/plantgenome2017.10.0090 (PMC12962448; doi:10.3835/plantgenome2017.10.0090)
Supplement: Supplementary file 1 [file TPG2-11-170090-s001.pdf]

Genomic Prediction in a Multiploid Crop: Genotype by Environment Interaction and Allele Dosage Effects on Predictive Ability in Banana

Moses Nyine, Brigitte Uwimana, Nicolas Blavet, Eva Hřibová, Helena Vanrespaille, Michael Batte, Violet Akech, Allan Brown, Jim Lorenzen,[&] Rony Swennen, Jaroslav Doležel[*]

M. Nyine, Faculty of Science, Palacký University, 77146, Olomouc, Czech Republic; M. Nyine, B. Uwimana, M. Batte, V. Akech, J. Lorenzen, International Institute of Tropical Agriculture, 7878, Kampala, Uganda; M. Nyine, N. Blavet, E. Hřibová, J. Doležel, Institute of Experimental Botany, Centre of the Region Haná for Biotechnological and Agricultural Research, CZ-78371, Olomouc, Czech Republic; H. Vanrespaille, R. Swennen, Laboratory of Tropical Crop Improvement, Division of Crop Biotechnics, Katholieke Universiteit 2455, 3001 Leuven, Belgium; R. Swennen, A. Brown, International Institute of Tropical Agriculture, 10, Arusha, Tanzania; [&]Current address: Bill & Melinda Gates Foundation, 23350, Seattle, USA. *Corresponding author (dolezel@ueb.cas.cz).

Supplemental Table S1: List of banana genotypes used in genomic predictions (Nyine et al. 2017)

S/No	Genotype name	Female parent	Male parent	Ploidy
1	Enzirabahima			Triploid
2	Kabucuragye			Triploid
3	Tereza			Triploid
4	Enyeru			Triploid
5	Nakayonga			Triploid
6	Namwezi			Triploid
7	Entukura			Triploid
8	Nakasabira			Triploid
9	Nakawere			Triploid
10	Nante			Triploid
11	Kazirakwe			Triploid
12	Nfuuka			Triploid
13	Calcutta 4			Diploid
14	1201K-1	Nakawere	Calcutta 4	Tetraploid
15	917K-2	Enzirabahima	Calcutta 4	Tetraploid
16	660K-1	Enzirabahima	Calcutta 4	Tetraploid
17	1438K-1	Entukura	Calcutta 4	Tetraploid
18	222K-1	Nfuuka	Calcutta 4	Tetraploid
19	376K-7	Nante	Calcutta 4	Tetraploid
20	365K-1	Kabucuragye	Calcutta 4	Tetraploid
21	401K-1	Entukura	Calcutta 4	Tetraploid
22	2180K-6			Diploid
23	8075-7	SH3362	Calcutta 4	Diploid
24	7197-2	SH3362	Long Tavoy	Diploid
25	SH3142	SH1734	Pisang Jari Buaya	Diploid
26	SH3362	SH3217	SH3142	Diploid
27	SH3217	SH2095	SH2766	Diploid
28	5610S-1	Kabucuragye	7197-2	Diploid
29	9128-3	Tjau lagada	Pisang lilin	Diploid
30	1968-2	Who-gu	Calcutta 4	Triploid
31	861S-1	Namwezi	Calcutta 4	Diploid
32	cv. Rose			Diploid

33	Pisang Lilin			Diploid
34	Kokopo			Diploid
35	Long Tavoy			Diploid
36	*M. a. M. a. malaccensis 250*			Diploid
37	28165S-1	1201K-1	1968-2	Triploid
38	25583S-2	1201K-1	5610S-1	Triploid
39	26660S-1	1201K-1	5610S-1	Triploid
40	28434S-9	1201K-1	5610S-1	Triploid
41	17503S-3	1201K-1	7197-2	Triploid
42	16242S-1	1201K-1	8075-7	Triploid
43	12479S-1	1201K-1	9128-3	Triploid
44	12479S-13	1201K-1	9128-3	Triploid
45	26317S-1	1201K-1	9128-3	Triploid
46	27262S-1	1201K-1	9128-3	Triploid
47	27262S-3	1201K-1	9128-3	Triploid
48	27770S-20	1201K-1	cv. Rose	Triploid
49	27770S-4	1201K-1	cv. Rose	Triploid
50	27935S-1	1201K-1	cv. Rose	Triploid
51	27960S-1	1201K-1	cv. Rose	Triploid
52	28036S-11	1201K-1	cv. Rose	Triploid
53	28036S-2	1201K-1	cv. Rose	Triploid
54	28164S-3	1201K-1	cv. Rose	Triploid
55	28246S-4	1201K-1	cv. Rose	Triploid
56	28246S-7	1201K-1	cv. Rose	Triploid
57	27935S-7	1201K-1	cv. Rose	Triploid
58	26363S-1	1201K-1	Kokopo	Triploid
59	26075S-6	1201K-1	Long Tavoy	Triploid
60	26075S-7	1201K-1	Long Tavoy	Triploid
61	26075S-8	1201K-1	Long Tavoy	Triploid
62	27346S-2	1201K-1	*M. a. malaccensis 250*	Triploid
63	27346S-4	1201K-1	*M. a. malaccensis 250*	Triploid
64	27437S-1	1201K-1	*M. a. malaccensis 250*	Triploid
65	27579S-1	1201K-1	*M. a. malaccensis 250*	Triploid
66	27579S-3	1201K-1	*M. a. malaccensis 250*	Triploid

67	28030S-2	1201K-1	*M. a. malaccensis 250*	Triploid
68	28030S-6	1201K-1	*M. a. malaccensis 250*	Triploid
69	28071S-1	1201K-1	*M. a. malaccensis 250*	Triploid
70	28465S-2	1201K-1	*M. a. malaccensis 250*	Triploid
71	28465S-21	1201K-1	*M. a. malaccensis 250*	Triploid
72	28479S-2	1201K-1	*M. a. malaccensis 250*	Triploid
73	26337S-22A	1201K-1	SH3217	Triploid
74	26337S-40	1201K-1	SH3217	Triploid
75	26840S-7	1201K-1	SH3362	Diploid
76	26315S-1	1201K-1	SH3142	Triploid
77	12419S-13	1201K-1	SH3217	Triploid
78	26337S-11A	1201K-1	SH3217	Triploid
79	26337S-2	1201K-1	SH3217	Triploid
80	26337S-34	1201K-1	SH3217	Triploid
81	26337S-37	1201K-1	SH3217	Triploid
82	26337S-39	1201K-1	SH3217	Triploid
83	26337S-43	1201K-1	SH3217	Triploid
84	28263S-2	1201K-1	SH3217	Triploid
85	12618S-1	1201K-1	SH3362	Triploid
86	26316S-7	1201K-1	SH3362	Triploid
87	26840S-10	1201K-1	SH3362	Triploid
88	25328S-3	1438K-1	1537K-1	Triploid
89	24948S-10	1438K-1	5610S-1	Triploid
90	24948S-13	1438K-1	5610S-1	Triploid
91	24948S-24	1438K-1	5610S-1	Triploid
92	24948S-9	1438K-1	5610S-1	Triploid
93	26060S-1	1438K-1	9128-3	Triploid
94	13573S-1	1438K-1	9719-7	Triploid
95	27914S-1	1438K-1	cv. Rose	Triploid
96	27914S-13	1438K-1	cv. Rose	Triploid
97	28095S-1	1438K-1	cv. Rose	Triploid
98	27264S-2	1438K-1	cv. Rose	Diploid
99	27914S-24	1438K-1	cv. Rose	Triploid
100	27914S-26	1438K-1	cv. Rose	Triploid

27914S-3	1438K-1	cv. Rose	Triploid
25474S-1	1438K-1	Kokopo	Triploid
26369S-4	1438K-1	Long Tavoy	Triploid
28481S-1	1438K-1	*M. a. malaccensis 250*	Triploid
28561S-2	1438K-1	*M. a. malaccensis 250*	Triploid
26725S-1	1438K-1	SH3362	Triploid
25499S-7	1438K-1	SH3142	Triploid
26039S-2	1438K-1	SH3217	Triploid
26466S-2	1977K-1	5610S-1	Triploid
26466S-5	1977K-1	5610S-1	Triploid
22598S-2	365K-1	1201K-1	Triploid
14539S-4	365K-1	660K-1	Triploid
9750S-13	401K-1	9128-3	Triploid
25031S-1	5610S-1	2180K-6	Diploid
25031S-15	5610S-1	2180K-6	Diploid
25031S-16	5610S-1	2180K-6	Diploid
25031S-17	5610S-1	2180K-6	Diploid
25031S-19	5610S-1	2180K-6	Diploid
25031S-27	5610S-1	2180K-6	Diploid
25031S-33	5610S-1	2180K-6	Diploid
25031S-34	5610S-1	2180K-6	Diploid
25031S-7	5610S-1	2180K-6	Diploid
24583S-2	660K-1	5610S-1	Triploid
26260S-3	660K-1	5610S-1	Triploid
13284S-1	660K-1	9128-3	Triploid
25371S-2	660K-1	9128-3	Triploid
9187S-8	660K-1	9128-3	Triploid
26709S-1	660K-1	Calcutta 4	Triploid
27713S-1	660K-1	*M. a. malaccensis 250*	Triploid
27825S-4	660K-1	*M. a. malaccensis 250*	Triploid
27873S-18	660K-1	*M. a. malaccensis 250*	Triploid
27873S-38	660K-1	*M. a. malaccensis 250*	Triploid
27873S-4	660K-1	*M. a. malaccensis 250*	Triploid
27873S-5	660K-1	*M. a. malaccensis 250*	Triploid

135	28188S-2	660K-1	*M. a. malaccensis 250*	Triploid
136	25623S-11	8817S-1	917K-2	Triploid
137	28492S-1	917K-2	1968-2	Triploid
138	26998S-1	917K-2	2180K-6	Triploid
139	27074S-1	917K-2	2180K-6	Triploid
140	25117S-1	917K-2	5610S-1	Triploid
141	25117S-2	917K-2	5610S-1	Triploid
142	25117S-3	917K-2	5610S-1	Triploid
143	25508S-1	917K-2	5610S-1	Triploid
144	25628S-11	917K-2	5610S-1	Triploid
145	26815S-3	917K-2	5610S-1	Triploid
146	26815S-8	917K-2	5610S-1	Triploid
147	26815S-9	917K-2	5610S-1	Triploid
148	26990S-10	917K-2	5610S-1	Triploid
149	26990S-11	917K-2	5610S-1	Triploid
150	26990S-4	917K-2	5610S-1	Triploid
151	27073S-1	917K-2	5610S-1	Triploid
152	27744S-1	917K-2	5610S-1	Triploid
153	12949S-2	917K-2	7197-2	Triploid
154	25909S-3	917K-2	7197-2	Triploid
155	25089S-4	917K-2	861S-1	Triploid
156	19798S-2	917K-2	9128-3	Triploid
157	24434S-3	917K-2	9128-3	Triploid
158	25435S-11	917K-2	9128-3	Triploid
159	25435S-4	917K-2	9128-3	Triploid
160	25737S-1	917K-2	9128-3	Triploid
161	26288S-4	917K-2	9128-3	Triploid
162	26975S-1	917K-2	9128-3	Triploid
163	26975S-2	917K-2	9128-3	Triploid
164	7798S-2	917K-2	9128-3	Triploid
165	27184S-4	917K-2	cv. Rose	Triploid
166	27885S-9	917K-2	cv. Rose	Triploid
167	27184S-8	917K-2	cv. Rose	Triploid
168	27494S-12	917K-2	cv. Rose	Triploid

169	27494S-4	917K-2	cv. Rose	Triploid
170	27494S-5	917K-2	cv. Rose	Triploid
171	28068S-9	917K-2	cv. Rose	Triploid
172	27184S-6	917K-2	cv. Rose	Triploid
173	27885S-1	917K-2	cv. Rose	Triploid
174	24410S-2	917K-2	Kokopo	Triploid
175	25680S-11	917K-2	Long Tavoy	Triploid
176	25680S-13	917K-2	Long Tavoy	Triploid
177	27261S-1	917K-2	*M. a. malaccensis 250*	Triploid
178	27261S-10	917K-2	*M. a. malaccensis 250*	Triploid
179	27261S-11	917K-2	*M. a. malaccensis 250*	Triploid
180	27334S-5	917K-2	*M. a. malaccensis 250*	Triploid
181	27401S-1	917K-2	*M. a. malaccensis 250*	Triploid
182	27524S-12A	917K-2	*M. a. malaccensis 250*	Triploid
183	27524S-12B	917K-2	*M. a. malaccensis 250*	Triploid
184	27524S-22	917K-2	*M. a. malaccensis 250*	Triploid
185	27524S-30	917K-2	*M. a. malaccensis 250*	Triploid
186	27833S-10	917K-2	*M. a. malaccensis 250*	Triploid
187	27833S-13	917K-2	*M. a. malaccensis 250*	Triploid
188	27886S-5	917K-2	*M. a. malaccensis 250*	Triploid
189	28033S-14	917K-2	*M. a. malaccensis 250*	Triploid
190	28033S-15	917K-2	*M. a. malaccensis 250*	Triploid
191	28033S-18	917K-2	*M. a. malaccensis 250*	Triploid
192	28033S-23	917K-2	*M. a. malaccensis 250*	Triploid
193	28033S-3	917K-2	*M. a. malaccensis 250*	Triploid
194	28060S-8	917K-2	*M. a. malaccensis 250*	Triploid
195	28200S-3	917K-2	*M. a. malaccensis 250*	Triploid
196	28257S-1	917K-2	*M. a. malaccensis 250*	Triploid
197	28257S-2	917K-2	*M. a. malaccensis 250*	Triploid
198	28257S-4	917K-2	*M. a. malaccensis 250*	Triploid
199	28432S-19	917K-2	*M. a. malaccensis 250*	Triploid
200	28432S-20	917K-2	*M. a. malaccensis 250*	Triploid
201	28432S-3	917K-2	*M. a. malaccensis 250*	Triploid
202	28780S-1	917K-2	*M. a. malaccensis 250*	Triploid

26874S-5	917K-2	SH3362	Triploid
12468S-18	917K-2	SH3217	Triploid
12477S-13	917K-2	SH3217	Triploid
8386S-19	917K-2	SH3217	Triploid
13522S-5	917K-2	SH3362	Triploid
25974S-?	917K-2	SH3362	Triploid
25974S-19	917K-2	SH3362	Triploid
25974S-21	917K-2	SH3362	Triploid
25974S-30	917K-2	SH3362	Triploid
25974S-35	917K-2	SH3362	Triploid
26666S-1	917K-2	SH3362	Triploid
28476S-7	917K-2	SH3362	Triploid
9494S-10	917K-2	SH3362	Triploid
16457S-2	Entukura	365K-1	Triploid
26540S-182	Entukura	8075-7	Diploid
28260S-2	Enzirabahima	Calcutta 4	Triploid
21086S-1	Kazirakwe	7197-2	Triploid
28073S-1	Namwezi	7197-2	Triploid
25356S-1	Tereza	7197-2	Triploid
HB	unknown	unknown	Triploid
HJ	unknown	unknown	Triploid
HX	unknown	unknown	Triploid
26337S-11B	1201K-1	SH3217	Triploid
16285S-13	Calcutta 4	660K-1	Diploid
26337S-22B	1201K-1	SH3217	Triploid
16285S-3	Calcutta 4	660K-1	Diploid
26337S-28	1201K-1	SH3217	Triploid
25066S-1	1438K-1	Kokopo	Triploid
16285S-6	Calcutta 4	660K-1	Diploid
25066S-2	1438K-1	Kokopo	Triploid
16285S-8	Calcutta 4	660K-1	Diploid
25974S-11	917K-2	SH3362	Triploid
25974S-15	917K-2	SH3362	Triploid
25457S-1	1438K-1	Kokopo	Triploid

16191S-6	Calcutta 4	917K-2	Diploid
24797S-7	917K-2	Kokopo	Triploid
25102S-1	917K-2	Kokopo	Triploid
28452S-11	Nakasabira	Calcutta 4	Triploid
28033S-9	917K-2	*M. a. malaccensis 250*	Triploid
25974S-13	917K-2	SH3362	Triploid
28256S-1	917K-2	cv. Rose	Triploid
25974S-17	917K-2	SH3362	Tetraploid
12468S-6	917K-2	SH3217	Triploid
27914S-11	1438K-1	cv. Rose	Triploid
27914S-18	1438K-1	cv. Rose	Triploid
27914S-21	1438K-1	cv. Rose	Triploid
27914S-22	1438K-1	cv. Rose	Triploid
27914S-6	1438K-1	cv. Rose	Triploid
27914S-7	1438K-1	cv. Rose	Triploid
27914S-8	1438K-1	cv. Rose	Triploid
27873S-12	660K-1	*M. a. malaccensis 250*	Triploid
27873S-14	660K-1	*M. a. malaccensis 250*	Triploid
27873S-17	660K-1	*M. a. malaccensis 250*	Triploid
27873S-33	660K-1	*M. a. malaccensis 250*	Triploid
27873S-37	660K-1	*M. a. malaccensis 250*	Triploid
27873S-7	660K-1	*M. a. malaccensis 250*	Triploid
26224S-3	1201K-1	SH3362	Triploid
26840S-9	1201K-1	SH3362	Triploid
26316S-14	1201K-1	SH3362	Triploid
26224S-2	1201K-1	SH3362	Triploid
26840S-5	1201K-1	SH3362	Triploid
25653S-3	1201K-1	SH3142	Triploid
26315S-3	1201K-1	SH3142	Triploid
28528S-1	1201K-1	Kokopo	Triploid
26369S-8	1438K-1	Long Tavoy	Triploid
26530S-1	1438K-1	SH3362	Triploid
27528S-1	1438K-1	*M. a. malaccensis 250*	Triploid
27915S-3	1438K-1	*M. a. malaccensis 250*	Triploid

28561S-5	1438K-1	*M. a. malaccensis 250*	Triploid
27915S-2	1438K-1	*M. a. malaccensis 250*	Triploid
28974S-11	1438K-1	*M. a. malaccensis 250*	Triploid
28974S-15	1438K-1	*M. a. malaccensis 250*	Triploid
28974S-22	1438K-1	*M. a. malaccensis 250*	Triploid
28974S-29	1438K-1	*M. a. malaccensis 250*	Triploid
29114S-1	5610S-1	*M. a. malaccensis 250*	Diploid
29114S-14	5610S-1	*M. a. malaccensis 250*	Triploid
29114S-19	5610S-1	*M. a. malaccensis 250*	Triploid
29114S-24	5610S-1	*M. a. malaccensis 250*	Triploid
27873S-26	660K-1	*M. a. malaccensis 250*	Triploid
27873S-31	660K-1	*M. a. malaccensis 250*	Triploid
29165S-5	660K-1	*M. a. malaccensis 250*	Triploid
28506S-1	Entukura	Calcutta 4	Triploid
29364S-2	Namwezi	cv. Rose	Tetraploid
28077S-5	Nfuuka	8075-7	Triploid
28164S-15	1201K-1	cv. Rose	Triploid
29285S-20	1201K-1	cv. Rose	Triploid
26337S-32	1201K-1	SH3217	Triploid
27684S-5	1201K-1	SH3362	Triploid
24948S-12	1438K-1	5610S-1	Triploid
24948S-21	1438K-1	5610S-1	Triploid
24948S-27	1438K-1	5610S-1	Triploid
29586S-4	1438K-1	5610S-1	Triploid
24948S-22	1438K-1	5610S-1	Triploid
24948S-2	1438K-1	5610S-1	Triploid
24948S-29	1438K-1	5610S-1	Triploid
26820S-1	917K-2	1968-2	Triploid
25474S-5	917K-2	861S-1	Triploid
25974S-18	917K-2	SH3362	Triploid
28476S-8	917K-2	SH3362	Triploid
25974S-31	917K-2	SH3362	Triploid
29275S-1	Enzirabahima	*M. a. malaccensis 250*	Tetraploid
29275S-4	Enzirabahima	*M. a. malaccensis 250*	Tetraploid

305	29275S-5	Enzirabahima	*M. a. malaccensis 250*	Tetraploid
306	29636S-1	Tereza	7197-2	Tetraploid
307	28776S-2	Tereza	8075-7	Triploid

Supplemental Table S2: Comparison of average correlation for five-fold cross validations between the predicted and observed phenotypes across all models fitted with data from either low input (GS1) or high input (GS2) fields and 10807 bi-allelic SNP markers

Trait category	Traits	BRR		BL		BayesA		BayesB		BayesC		RKHS_P		RKHS_M		RKHS_PM	
		GS1	GS2	GS1	GS2	GS1	GS2	GS1	GS2	GS1	GS2	GS1	GS2	GS1	GS2	GS1	GS2
Plant stature	Plant height	0.54	0.46	0.55	0.45	0.54	0.45	0.54	0.44	0.54	0.45	0.42	0.40	0.55	0.44	0.54	0.48
	Plant girth	0.60	0.52	0.6	0.51	0.6	0.51	0.60	0.52	0.60	0.51	0.44	0.40	0.60	0.51	0.55	0.50
Suckering behaviour	Total number of suckers	0.16	0.17	0.17	0.20	0.17	0.20	0.16	0.19	0.15	0.19	0.12	0.12	0.17	0.18	0.16	0.17
	Height of tallest sucker at flowering	0.28	0.18	0.30	0.20	0.28	0.18	0.27	0.20	0.26	0.20	0.27	0.24	0.28	0.19	0.30	0.24
	Height of tallest sucker at harvesting	0.27	0.26	0.26	0.28	0.28	0.25	0.28	0.24	0.27	0.25	0.28	0.29	0.26	0.26	0.29	0.32
Black leaf streak	Number of standing leaves at flowering	0.36	0.42	0.37	0.40	0.37	0.42	0.43	0.40	0.36	0.41	0.17	0.19	0.37	0.41	0.29	0.34
	Index of non-spotted leaves	0.35	0.42	0.35	0.42	0.34	0.43	0.34	0.43	0.34	0.43	0.22	0.22	0.35	0.42	0.32	0.36
Fruit bunch	Days to fruit maturity	0.47	0.42	0.47	0.42	0.47	0.42	0.47	0.42	0.46	0.42	0.44	0.41	0.47	0.42	0.49	0.44
	Bunch mass	0.63	0.61	0.62	0.61	0.62	0.62	0.64	0.62	0.64	0.62	0.41	0.43	0.61	0.61	0.52	0.55
	Number of hands	0.60	0.62	0.59	0.62	0.59	0.63	0.60	0.62	0.59	0.62	0.34	0.39	0.59	0.62	0.48	0.53
	Number of fruits	0.47	0.51	0.47	0.53	0.47	0.52	0.47	0.52	0.47	0.52	0.25	0.33	0.45	0.52	0.35	0.45
Fruit filling	Fruit length	0.65	0.64	0.65	0.64	0.65	0.64	0.67	0.65	0.67	0.65	0.50	0.48	0.64	0.64	0.59	0.59
	Fruit circumference	0.67	0.66	0.67	0.67	0.66	0.66	0.70	0.69	0.70	0.69	0.40	0.42	0.65	0.66	0.57	0.60
	Fruit diameter	0.67	0.63	0.67	0.68	0.66	0.67	0.70	0.71	0.70	0.71	0.39	0.40	0.65	0.67	0.57	0.59
	Pulp diameter	0.67	0.68	0.67	0.69	0.66	0.68	0.70	0.72	0.70	0.72	0.39	0.41	0.65	0.67	0.57	0.60

The values under GS1 column are the correlations between predicted and observed phenotype (predictive ability) in GS2 when GS1 data were used to fit the model and vice versa for GS2 column.

Supplemental Table S3: Comparison of predictive ability of BayesB model fitted with parents' data and progeny's data using bi-allelic and allele dosage SNP markers

		Parents model			Progeny model		
Trait category	Traits	BA-SNP	AD-SNP	LIP	BA-SNP	AD-SNP	LIP
Plant stature	Plant height	0.36	0.18	-50.0	0.77	0.51	-33.8
	Plant girth	0.39	0.05	-87.2	0.80	0.43	-46.3
Suckering behaviour	Total number of suckers	0.13	0.06	-53.8	0.39	0.22	-43.6
	Height of tallest sucker at flowering	0.23	0.12	-47.8	0.50	0.37	-26.0
	Height of tallest sucker at harvesting	0.19	-0.15	-178.9	0.43	-0.03	-107.0
Black leaf streak	Number of standing leaves at flowering	0.31	0.20	-35.5	0.43	0.46	7.0
	Index of non-spotted leaves	0.39	0.33	-15.4	0.85	0.77	-9.4
Fruit bunch	Days to fruit maturity	0.39	0.32	-17.9	0.77	0.66	-14.3
	Bunch mass	0.50	0.17	-66.0	0.66	0.08	-87.9
	Number of hands	0.45	0.03	-93.3	0.86	0.48	-44.2
	Number of fruits	0.31	0.10	-67.7	0.77	0.36	-53.2
Fruit filling	Fruit length	0.59	0.23	-61.0	0.78	0.22	-71.8
	Fruit circumference	0.49	0.17	-65.3	0.65	0.62	-4.6
	Fruit diameter	0.42	0.22	-47.6	0.66	0.65	-1.5
	Pulp diameter	0.49	0.23	-53.1	0.66	0.68	3.0

LIP = 100*((prediction with AD-SNP – prediction with BA-SNP)/prediction with BA-SNP)

Supplemental Table S4: Effect of ploidy level and allele dosage on the predictive ability of BayesB model fitted with environment averaged phenotype data

		Bi-allelic SNP			Allele dosage SNP		
Trait category	Traits	Tetraploid	Triploid	Diploid	Tetraploid	Triploid	Diploid
Plant stature	Plant height	0.04	0.37	0.71	-0.54	0.10	0.09
	Plant girth	0.02	0.38	0.72	-0.02	-0.06	-0.46
Suckering behaviour	Total number of suckers	-0.17	0.07	0.34	-0.48	-0.04	0.01
	Height of tallest sucker at flowering	0.19	0.31	0.32	-0.42	0.01	-0.13
	Height of tallest sucker at harvesting	0.06	0.20	0.57	-0.15	-0.03	-0.17
Black leaf streak	Number of standing leaves at flowering	0.19	0.40	0.38	0.05	0.09	-0.12
	Index of non-spotted leaves	-0.09	0.44	0.70	-0.30	0.12	0.31
Fruit bunch	Days to fruit maturity	0.01	0.46	0.56	0.01	0.06	0.21
	Bunch mass	0.15	0.39	0.73	0.03	0.03	-0.50
	Number of hands	0.33	0.44	0.70	0.48	0.08	0.05
	Number of fruits	0.50	0.37	0.57	-0.21	0.03	0.08
Fruit filling	Fruit length	-0.10	0.54	0.86	0.25	0.06	-0.21
	Fruit circumference	-0.15	0.43	0.79	0.35	0.05	-0.25
	Fruit diameter	-0.45	0.39	0.77	0.53	0.11	-0.15
	Pulp diameter	-0.39	0.41	0.79	0.60	-0.05	-0.23

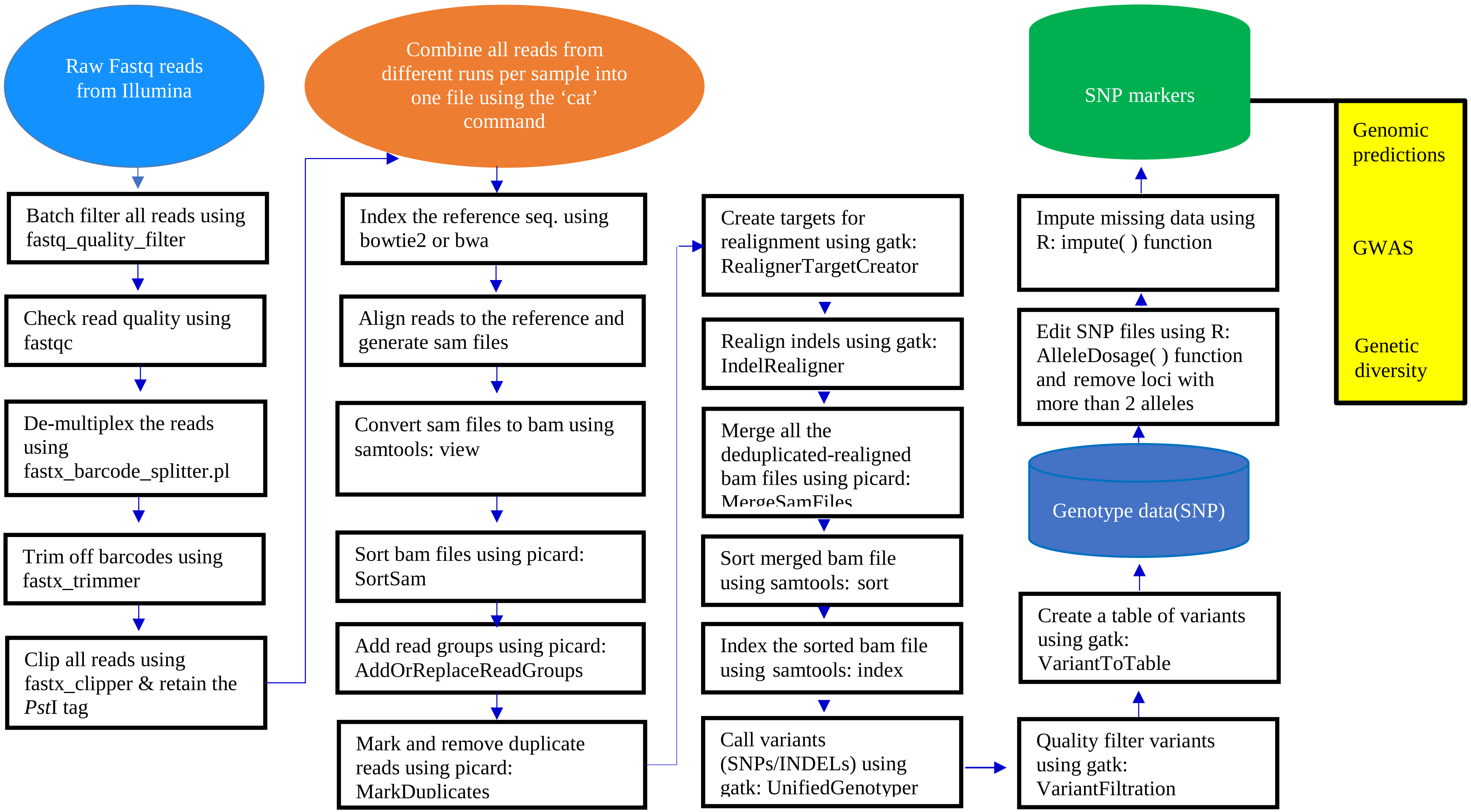

Supplemental Fig. S1: Workflow used to analyse the genotyping by sequencing (GBS) reads to generate SNP marker data used in genomic predictions
